# Overexpression of sphingosine kinase 1 is associated with salivary gland carcinoma progression and might be a novel predictive marker for adjuvant therapy

**DOI:** 10.1186/1471-2407-10-495

**Published:** 2010-09-16

**Authors:** Guanglin Liu, Haiqing Zheng, Zhibing Zhang, Zhiqiang Wu, Huaping Xiong, Jun Li, Libing Song

**Affiliations:** 1 State Key Laboratory of Oncology in Southern China, Department of Experimental Research, Cancer Center, Sun Yat-sen University, Guangzhou, Guangdong, China; 2 Department of Rehabilitation Medicine, The Third Affiliated Hospital, Sun Yat-sen University, Guangzhou, Guangdong, China; 3 Department of Biochemistry, Zhongshan School of Medicine, Sun Yat-sen University, Guangzhou, Guangdong, China; 4 Department of Microbiology, Zhongshan School of Medicine, Sun Yat-sen University, Guangzhou, Guangdong, China

## Abstract

**Background:**

Overexpression of sphingosine kinase-1 (SPHK1) has been demonstrated to be associated with the development and progression in various types of human cancers. The current study was to characterize the expression of SPHK1 in salivary gland carcinomas (SGC) and to investigate the association between SPHK1 expression and progression of SGC.

**Methods:**

The expression of SPHK1 was examined in 2 normal salivary gland tissues, 8 SGC tissues of various clinical stages, and 5 pairs of primary SGC and adjacent salivary gland tissues from the same patient, using real-time PCR and western blot analysis. Furthermore, the SPHK1 protein expression was analyzed in 159 clinicopathologically characterized SGC cases by immunohistochemistry. Statistical analyses were performed to determine the prognostic and diagnostic associations.

**Results:**

SPHK1 expression was found to be markedly upregulated in SGC tissues than that in the normal salivary gland tissues and paired adjacent salivary gland tissues, at both mRNA and protein levels. Statistical analysis revealed a significant correlation of SPHK1 expression with the clinical stage (*P *= 0.005), T classification (*P *= 0.017), N classification (*P *= 0.009), M classification (*P *= 0.002), and pathological differentiation (*P *= 0.013). Patients with higher SPHK1 expression had shorter overall survival time, whereas patients with lower SPHK1 expression had better survival. Importantly, patients in the group without adjuvant therapy who exhibited high SPHK1 expression had significantly lower overall survival rates compared with those with low SPHK1 expression. Moreover, multivariate analysis suggested that SPHK1 expression might be an independent prognostic indicator for the survival of SGC patients.

**Conclusions:**

Our results suggest that SPHK1 expression is associated with SGC progression, and might represent as a novel and valuable predictor for adjuvant therapy to SGC patients.

## Background

Salivary gland carcinoma (SGC), a relatively rare neoplasm, accounts for 0.5% of all malignancies and approximately 3%-5% of all head and neck cancers worldwide [1,2]. SGC is a special type of cancer owing to the wide variation in its histological and clinical features [3]. According to the WHO classification, SGC contains 24 different entities and consists of 4 main histopathological types, namely, mucoepidermoid carcinoma (MEC), adenoid cystic carcinoma (ACC), acinic cell carcinoma (AcCC), and salivary duct carcinoma (SDC). All other types of SGC occur less frequently or rarely [1,3]. Although advances have been made in developing more elegant techniques, new chemotherapeutic agents, and radiotherapy, the prognosis of advanced SGC has not been significantly improved. The overall survival at 5 and 10 years for SGC patients is 92% and 90%, respectively; however, all the patients who had clinical stage III or IV disease at diagnosis subsequently died [4]. Further, more than 5% of patients suffered a recurrence at the primary site and/or distant metastases, the latter of which are not amenable to surgery or radiotherapy [5]. Therefore, it will be of great clinical value to identify effective early markers for the diagnosis and prognosis of the disease and also novel therapeutic targets.

Mounting evidence suggests that dysregulation of lipogenesis/lipid metabolism is closely associated with the initiation and progression of various types of cancer. For instance, sphingosine-1-phosphate (S1P), a bioactive lipid mediator, has been demonstrated to play critical roles in fundamental biological processes, such as proliferation, survival, migration, angiogenesis, and invasion [6-11]. Consistent with this, the super-activation or upregulation of SPHK1, which catalyzes the phosphorylation of sphingosine to S1P, is shown to be involved in carcinogenesis. Overexpression of SPHK1 in NIH3T3 fibroblasts enhances cell proliferation and the ability of anchorage-independent growth, as well as tumorigenicity, in NOD/SCID mice [12]. Further, silencing the endogenous expression of SPHK1 in glioblastoma cells and breast cancer cells leads to cell cycle arrest. Inhibition of SPHK1 using a dominant-negative form of SPHK1 dramatically decreased tumor formation in nude mice [13]. Blocking SPHK1 activity by using its inhibitors, such as camptothecin or docetaxel, has been demonstrated to suppress tumor growth and to reduce tumor occurrence and metastases in nude mice [14,15]. Further, several studies have shown that ectopic expression of SPHK1 can protect cancer cells against apoptosis in response to pro-apoptotic stimuli, such as treatment with TNF-α, ionizing radiation, or anti-cancer drugs, through multiple pathways [16,17]. It has been reported that the upregulation of SPHK1 impairs the effectiveness of chemotherapy in human PC-3 and LNCaP prostate cancer cell lines [18,19]. Bonhoure et al. demonstrated that ectopic expression of SPHK1 in HL-60 cells promoted resistance to treatments with doxorubicin and etoposide by reducing the production of ceramide [20]. Upregulation of SPHK1 protects LAMA84 erythromegakaryocytic cells from imatinib by blocking the release of cytochrome c and Smac/Diablo from mitochondria [21]. Indeed, the expression level of SPHK1 has been found to be frequently upregulated in various tumor types, including glioblastoma multiforme, intestinal adenoma, acute erythroleukemia, prostate cancer, colon cancer, and gastric cancer [22-26].

In the current study, we report, for the first time, the characterization of SPHK1 expression in SGC of various clinicopathological grades. We found that the expression of SPHK1 was significantly correlated with the clinical stage, TNM classification, and pathological differentiation of SGC. Statistical analysis revealed that SPHK1 expression represents a potentially useful independent biomarker for the prognosis of SGC patients and might serve as a valuable predictor for adjuvant therapy to SGC patients.

## Methods

### Patients and tissue specimens

This study was conducted on a total of 159 paraffin-embedded SGC samples, which were histopathologically and clinically diagnosed at the Sun Yat-sen University Cancer Center from 1995 to 2004. Fifty patients was received adjuvant radiation therapy, which doses ranged from 6 Gy as palliative single fraction to 60 Gy. Two human normal salivary gland tissues were obtained from patients with head and neck tumors during the surgical procedure of neck dissection. For the use of these clinical materials for research purposes, prior patient consent and approval from the Institutional Research Ethics Committee were obtained. Clinical and clinicopathological classification and staging were determined according to the criteria proposed by WHO classification [3]. Clinical information on the samples is summarized in Table 1. Five specimens of SGC tissues (Clinical stage II) and the matched adjacent noncancerous salivary gland tissues were frozen and stored in liquid nitrogen until further use.

**Table 1:** Clinicopathological characteristics of SGC patient samples

	Number of cases (%)
**Gender**	

Male	89 (56.0%)

Female	70 (44.0%)

**Age (years)**	

≥47	81 (50.9%)

< 47	78 (49.1%)

**Clinical Stage**	

I	16 (10.1%)

II	58 (36.5%)

III	39 (24.5%)

IV	46 (28.9%)

**T classification**	

T1	17 (10.7%)

T2	72 (45.3%)

T3	37 (23.3%)

T4	33 (20.8%)

**N classification**	

N0	118 (74.2%)

N1	21 (13.2%)

N2	20 (12.6%)

N3	0 (0.0%)

**M classification**	

No	113 (71.1%)

Yes	46 (28.9%)

**Pathologic Differentiation**	

Poor	101 (63.5%)

Moderate	4 (2.5%)

Well	54 (34.0%)

**Histological Types**	

MEC	37 (23.3%)

ACC	23 (14.5%)

AcCC	18 (11.3%)

SDC	18 (11.3%)

Other histological types	63 (39.6%)

**Vital status (at follow-up)**	

Alive	107 (67.3%)

Death (All Salivary Gland cancer-related)	52 (32.7%)

**Adjuvant Therapy**	

Yes	50 (31.5%)

No	109 (68.6%)

### RNA extraction and Real-time PCR

Total RNA from cells and primary tumor samples was extracted using the Trizol reagent (Invitrogen, Carlsbad, CA) according to the manufacturer's instruction. Real-time PCR was performed according to standard methods as described previously [26]. Sequences of the real-time PCR primers were designed using the Primer Express Software Version 2.0 and sequences are: *SPHK1 *forward primer: 5'-CTTGCAGCTC TTCCGGAGTC-3', SPHK1 reverse primer 5'-GCTCAGTGAGCATCAGCGTG-3', *SPHK1 *probe 5'-(FAM)CCCTTTTGGCTGAGGCTGAAATC TCC(TAMRA)-3'. Expression data were normalized to the geometric mean of housekeeping gene *GAPDH *to control the variability in expression levels (forward primer 5'-GACTCATGACCACAGTCCATGC-3', reverse primer 5'-AGAGGCAGGGATGATGTTCT G-3', and probe 5'-(FAM)CATCACTGC CACCCAGAAG ACTGTG(TAMRA)-3') and calculated as 2^-[(Ct of *SPHK1*) - (Ct of *GAPDH*)]^, where C_t _represents the threshold cycle for each transcript.

### Western blotting

Western blotting was performed according to standard methods as described previously [27], using rabbit anti-SPHK1 (1:1000; Abgent, San Diego, CA). The membranes were stripped and re-blotted with an anti-α-tubulin antibody (Sigma, Saint Louis, MI) as a loading control.

### Immunohistochemistry

Immunohistochemical analysis was performed to study altered protein expression in two human normal salivary gland tissues and 159 human salivary gland carcinomas tissues. The procedures were carried out similarly to previously described methods [26]. In brief, paraffin-embedded specimens were cut into 4-μm sections and baked at 60°C for 2 hrs, followed by deparaffinization with xylene and rehydrated. The sections were submerged into EDTA antigenic retrieval buffer and microwaved for antigenic retrieval, after which they were treated with 3% hydrogen peroxide in methanol to quench endogenous peroxidase activity, followed by incubation with 1% bovine serum albumin to block nonspecific binding. Sections were incubated with rabbit anti-SPHK1 (Cat# AP7237c; Abgent, San Diego, CA) overnight at 4°C. Normal goat serum was used as a negative control. After washing, tissue sections were treated with biotinylated anti-rabbit secondary antibody (Zymed, San Francisco, CA), followed by further incubation with streptavidin-horseradish peroxidase complex (Zymed, San Francisco, CA). Tissue sections were then immersed in DAB (3.3'-diaminobenzidine) and counterstained with 10% Mayer's hematoxylin, dehydrated and mounted.

The degree of immunostaining was reviewed and scored independently by 2 observers based on the proportion of positively stained tumor cells and intensity of staining. Tumor cell proportion was scored as follows: 0 (no positive tumor cells), 1 (<10% positive tumor cells), 2 (10-35% positive tumor cells), 3 (35-70% positive tumor cells), and 4 (>70% positive tumor cells). Staining intensity was graded according to the following criteria: 0 (no staining), 1 (weak staining = light yellow), 2 (moderate staining = yellow brown), and 3 (strong staining = brown). Staining index (SI) was calculated as the product of staining intensity score and the proportion of positive tumor cells. Using this method of assessment, we evaluated SPHK1 expression in benign salivary gland epithelia and malignant lesions by determining the SI, with scores of 0, 1, 2, 3, 4, 6, 8, 9 or 12. The cutoff value for high- and low-expression level was chosen on the basis of a measure of heterogeneity with the log-rank test statistical analysis with respect to overall survival. An optimal cutoff value was identified: an SI score of ≥6 was used to define tumors with high SPHK1 expression, an SI score of ≤4 but > 0 was used to indicate low SPHK1 expression, and SI score of = 0 was used to indicate no SPHK1 expression.

IHC staining for protein expression in tumor and normal tissues was quantitative analyzed with the AxioVision Rel.4.6 computerized image analysis system assisted with the automatic measurement program (Carl Zeiss, Oberkochen, Germany). Briefly, the stained sections were evaluated at 200× magnification, and ten representative staining fields of each section were analyzed to verify the Mean Optical Density (MOD), which represents the strength of staining signals as measured per positive pixels. The MOD data were statistically analyzed using Student's t-tests to compare the average MOD difference between different groups of tissues, and *P *< 0.05 was considered significant.

### Statistical analysis

All statistical analyses were carried out using the SPSS 13.0 statistical software package. The chi-square test was used to analyze the relationship between SPHK1 expression and clinicopathological characteristics. Bivariate correlations between study variables were calculated by Spearmans rank correlation coefficients. Survival curves were plotted by the Kaplan-Meier method and compared using the log-rank test. Survival data were evaluated using univariate and multivariate Cox regression analyses. *P *< 0.05 in all cases was considered statistically significant.

## Results

### Expression of SPHK1 is upregulated in SGC

**Figure 1:**
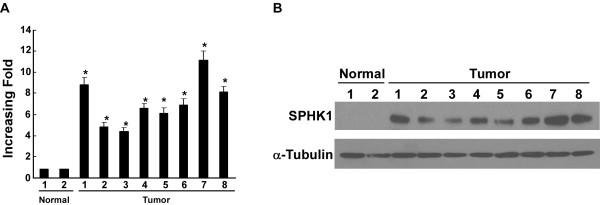
**SPHK1 expression is upregulated in SGC tissues**. **(A and B) **Expression analyses of SPHK1 mRNA and protein in 2 normal human salivary gland tissues and 8 SGC tissues by real-time PCR **(A) **and western blotting **(B)**. Data in **A **to **B **was performed three independent times with similar results. *, *P *< 0.05.

To investigate whether SPHK1 expression is upregulated in clinical SGC tissues, real-time PCR analysis and western blot analysis were performed in 2 normal salivary gland tissues and 8 fresh-frozen SGC tissues. As shown in Fig. 1A, real-time PCR results showed that all SGC tissues exhibited significantly higher levels of SPHK1 mRNA compared with normal salivary gland tissues. In parallel with the upregulation of mRNA, western blot analysis revealed that SPHK1 protein was overexpressed in all 8 SGC, whereas it was barely detectable in normal salivary gland tissues (Fig. 1B).

**Figure 2:**
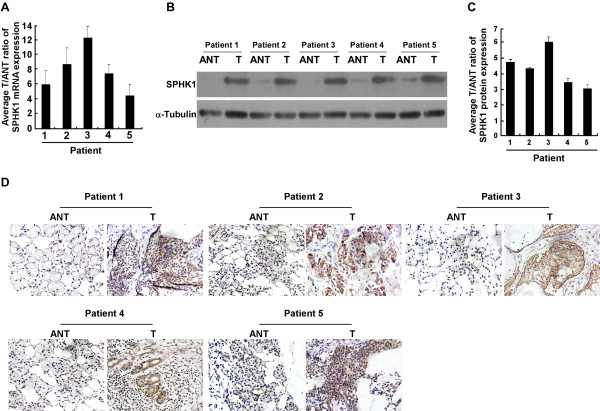
**Expression of SPHK1 is elevated in human primary SGC tissues compared with adjacent noncancerous tissues**. **(A) **Expression of SPHK1 mRNA in each of the primary SGC tissues (T) and SGC-adjacent noncancerous tissues (ANT) in the same patient, determined by real-time PCR. **(B and C) **Expression (B) and Quantification (C) of SPHK1 protein in each of the primary SGC tissues (T) and SGC-adjacent noncancerous tissues (ANT) in the same patient, determined by western blotting. **(D) **Expression of SPHK1 protein in each of the primary SGC tissues (T) and SGC-adjacent noncancerous tissues (ANT) in the same patient, determined by immunohistochemistry (IHC). Data in **A **to **D **was performed three independent times with similar results. *, *P *< 0.05.

Comparative analysis was conducted to examine the expression of SPHK1 in 5 pairs of primary SGC tissue and adjacent noncancerous tissue. Real-time PCR analysis revealed that SPHK1 mRNA was overexpressed in the 5 primary SGC samples compared with the paired adjacent noncancerous salivary gland tissues, the overexpression was as high as 12.5-fold in 1 of the 5 paired primary SGC tissues (Fig. 2A). Meanwhile, the expression of the SPHK1 protein was also found to be upregulated in all the 5 human primary SGC tissue samples as compared to the expression in their matched adjacent noncancerous tissues using western blot (Fig. 2B). Importantly, protein quantification of western blotting analysis (intensity) showed that all 5 tumors displayed a more than 3-fold increase in SPHK1 protein compared with the tissues adjacent to the tumors (Fig. 2C). The abovementioned result was further confirmed by immunohistochemistry (IHC) analyses (Fig. 2D). Taken together, our results suggest that SPHK1 is upregulated at both the mRNA and protein levels in SGC.

### Overexpression of SPHK1 protein in archived SGC samples

**Figure 3:**
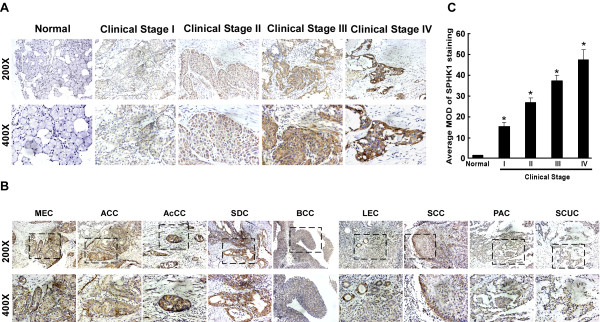
**SPHK1 protein is overexpressed in SGC histopathological sections as examined by immunohistochemistry**. **(A and B) **Representative images from immunohistochemical (IHC) analyses of normal human salivary gland tissues and 159 primary SGC specimens **(A)**, as well as 9 histological types **(B)**. **(C) **Statistical analyses of the average MOD of SPHK1 staining between normal salivary gland tissues (3 cases) and SGC specimens of different clinical stage (16 cases per stage). * *P *< 0.05

To further evaluate whether SPHK1 protein upregulation is linked to the clinical progression of SGC, IHC analysis was performed to examine the SPHK1 protein expression in 2 paraffin-embedded normal salivary gland tissue samples and 159 paraffin-embedded, archived SGC tissue samples, including 9 histological types of SGC. *i.e*. mucoepidermoid carcinoma (MEC), adenoid cystic carcinoma (ACC), acinar cell carcinoma(AcCC), salivary duct carcinoma(SDC), basal cell carcinoma(BCC), lymphoepithelial carcinoma(LEC), squamous cell carcinoma(SCC), papillary adenocarcinoma(PAC) and small cell undifferentiated carcinoma(SCUC). The immunohistochemical results are summarized in Table 1 and 2. SPHK1 protein was detected in 154 of the 159 (96.9%) cases. As shown in Fig. 3A and 3B, the expression of SPHK1 was upregulated in all the examined histological types of SGC compared with the normal salivary gland tissues. High levels of SPHK1 expression were present in areas containing the primary SGC cells, whereas SPHK1 was undetectable or only marginally detectable in the adjacent noncancerous tissues in all tumor sections and in normal salivary gland tissues. SPHK1 was mainly localized in the cytoplasm of primary cancer cells, which is consistent with previous reports on SPHK1 expression in other cancer types [22-26]. In order to avoid possible bias caused by selecting fields that have more cells or those that are heavily stained, the representative staining fields of each tumor sample were further analyzed to verify the mean optical density (MOD). Quantitative analysis indicated that the average MOD of SPHK1 staining in primary tumors of clinical stages I-IV were statistically significantly higher than those in normal salivary gland tissue (*P *< 0.001, Fig. 3C). Taken together, these observations suggested that SPHK1 protein is overexpressed in archived SGC samples.

**Table 2:** The expression of SPHK1 in Salivary Gland Cancer

Expression of SPHK1	
Negative	5 (3.1%)

Positive	154 (96.9%)

Low expression	69 (46.5%)

High expression	85 (53.5%)

### Increased expression of SPHK1 is correlated with the clinical features of SGC

The results of the immunohistochemical analyses of SPHK1 levels were further statistically analyzed to determine the relationship with the clinical features of SGC. As shown in Table 3, SPHK1 expression was significantly correlated with the clinical stage (*P *= 0.005), T classification (*P *= 0.017), N classification (*P *= 0.009), M classification (*P *= 0.002) and pathological differentiation (*P *= 0.013) of patients with SGC, whereas it was not associated with age, gender, or histological type. The above results were further analyzed using Spearman correlation analysis, which revealed that the Spearman correlation coefficients between SPHK1 expression levels and clinical stage, T classification, N classification, M classification, and pathological differentiation were 0.277 (*P *< 0.001), 0.239 (*P *= 0.020), 0.158 (*P *= 0.004), 0.234 (*P *= 0.003), and 0.245 (*P *= 0.020), respectively (Table 4). Taken together, these results indicate that the overexpression of SPHK1 is correlated with the clinical features of SGC.

**Table 3:** Correlation between SPHK1 expression and clinicopathologic characteristics of SGC

Characteristics		SPHK1		Chi-square test *P*-value
			
		Low or none No. cases (%)	High No. cases (%)	
Gender	Male	45 (67.2)	44 (47.8)	0.150
		
	Female	22 (32.8)	48 (52.2)	

Age (years)	≥ 47	34 (50.7)	47 (51.1)	0.966
		
	< 47	33 (49.3)	45 (48.9)	

Clinical Stage	I	10 (13.5)	6 (7.1)	0.005
		
	II	35 (47.3)	23 (27.1)	
		
	III	16 (21.6)	23 (27.1)	
		
	IV	13 (17.6)	33 (38.8)	

T classification	T1	10 (13.5)	7 (8.2)	0.017
		
	T2	40 (54.1)	32 (37.6)	
		
	T3	16 (21.6)	21 (24.7)	
		
	T4	8 (10.8)	25 (29.4)	

N classification	N0	60 (81.1)	58 (68.2)	0.009
		
	N1	9 (12.2)	12 (14.1)	
		
	N2	5 (6.8)	15 (17.6)	
		
	N3	0 (0.0)	0 (0.0)	

M classification	No	61 (80.6)	52 (64.1)	0.002
		
	Yes	13 (19.4)	33 (35.9)	

Pathologic Differentiation	Poor	57(35.8)	44(27.7)	0.013
		
	Moderate	4(2.5)	0(0.0)	
		
	Well	24(15.1)	30(18.9)	

Histological Types	MEC	15(20.3)	22(25.9)	0.945
		
	ACC	11(14.9)	12(14.1)	
		
	AcCC	9(12.2)	9(10.6)	
		
	SDC	9(12.2)	9(10.6)	
		
	Other histological types	30(40.5)	33(38.8)	

**Table 4:** Spearman correlation analysis between SPHK1 and clinical pathologic factors

Variables	SPHK1 expression level	
	
	Spearman Correlation	*P*-Value
Clinical staging	0.277	<0.001

T classification	0.239	0.020

N classification	0.158	0.004

M classification	0.234	0.003

Pathologic Differentiation	0.245	0.020

### SPHK1 expression is associated with the prognosis of patients with SGC

**Figure 4:**
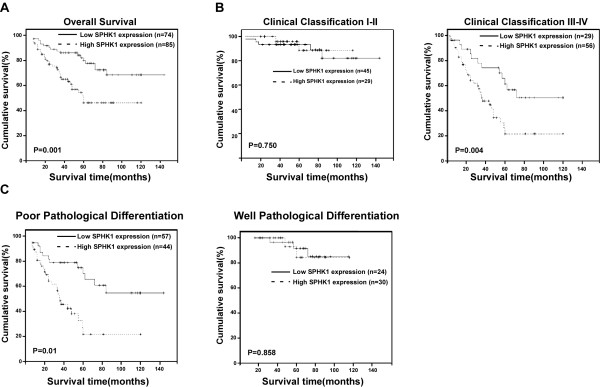
**Kaplan-Meier curves with univariate analyses (log-rank) for patients with low SPHK1 expression versus high SPHK1 expression tumors**. **(A) **Patients with low SPHK1 expression (bold line) had a cumulative 5-year survival rate of 93.4% (95% confidence interval, 0.854-0.914), compared to the 46.3% for patients with high SPHK1 expression (dotted line; 95% confidence interval, 0.369-0.573). **(B) **Statistical analysis of the difference between the tumors with high and low SPHK1 expression in the clinical stages I and II (left) and clinical stages III and IV (right) patient subgroups. **(C) **Statistical analysis of the difference between tumors with high and low SPHK1 expression in the poor pathologic differentiation (left) and well pathologic differentiation (right) patient subgroups. *P *values were calculated using the log-rank test.

Patient survival analysis were conducted and revealed that SPHK1 protein expression in SGC was significantly associated with the survival time of patients (*P *< 0.001), with a correlation coefficient of -0.363, clearly indicating that the expression of SPHK1 was inversely correlated with survival time (Table 5). Furthermore, Kaplan-Meier analysis and the log-rank test were used to evaluate the effects of clinicopathological characteristics, including age, gender, histological type, clinical stage, T classification, N classification, and M classification, in conjunction with SPHK1 protein expression, on patient survival. As shown in Fig. 4A, the length of survival time was significantly different between the patients with low and high SPHK1 expression (*P *= 0.001), with the high SPHK1 expression group having a shorter overall survival time. The cumulative 5-year survival rate was 93.4% (95% confidence interval, 0.854-0.914) in the low SPHK1 expression group, whereas it was only 46.3% (95% confidence interval, 0.369-0.573) in the high SPHK1 expression group. Furthermore, univariate and multivariate analyses were performed to determine whether the SPHK1 expression level is an independent prognostic factor of patient outcome. As shown in Table 4, clinical stage, N classification and pathologic classification, as well as SPHK1 expression, were identified as independent prognostic factors, indicating that SPHK1 might be a valuable marker for SGC patient diagnostic period.

**Table 5:** Univariate and multivariate analyses of various prognostic parameters in patients with SGC Cox-regression analysis

	Univariate analysis			Multivariate analysis		
	
	No. patients	*P*	Regression coefficient (SE)	*P*	Relative risk	95% confidence interval
**Clinical stage**						

I	16	<0.001	0.214(0.639)	0.003	1.894	1.244-2.884
					
II	58					
					
III	39					
					
IV	46					
					
**N classification**						

N0	118	<0.001	0.203(0.764)	<0.001	2.146	1.441-3.196
					
N1	21					
					
N2	20					
					
N3	0					

**Pathologic Differentiation**						

Poor	101	<0.001	0.168(0.358)	0.033	0.699	0.503-0.972
					
Moderate	4					
					
Well	54					

**Expression of SPHK1**						

Low expression	74	0.001	0.324(0.657)	0.042	1.929	1.023-3.636
					
High expression	85					

SPHK1 protein expression level in SGC significantly correlated with patient survival time (*P *< 0.001); the correlation coefficient was -0.363, indicating that higher levels of SPHK1 expression correlated with shorter survival time.

Moreover, we evaluated the prognostic value of SPHK1 expression in selected patient subgroups. Although we did not find any difference in the overall survival times of patients between the low and high SPHK1 expression groups in the early clinical subgroup (stages I-II, n = 74; log-rank, *P *= 0.75; Fig. 4B, left panel), the patients in the advanced disease group (stages III-IV) with tumors exhibiting high SPHK1 expression had significantly lower overall survival rates compared with those with a low level of SPHK1 expression (n = 85; log-rank, *P *= 0.004; Fig. 4B, right panel). Interestingly, statistical analysis revealed a significant difference between the curves of low and high SPHK1-expressing patients grouped by poor pathologic differentiation (n = 101; log-rank, *P *= 0.01; Fig. 4C, left panel), but no difference between the curves of low and high SPHK1-expressing patients grouped by well pathologic differentiation (n = 54; log-rank, *P *= 0.858; Fig. 4C, right panel). Taken together, our data suggest that SPHK1 might be a novel and potentially useful independent biomarker for the prognosis of patients with SGC.

### SPHK1 expression might be a valuable predictor for adjuvant therapy to SGC patients

**Figure 5:**
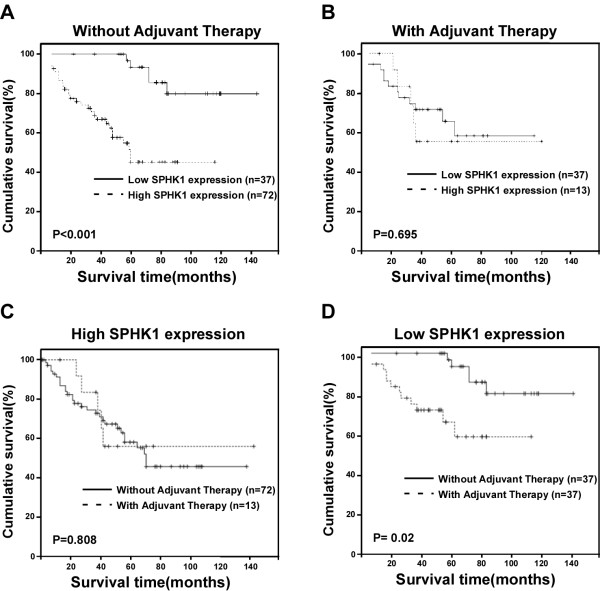
**Kaplan-Meier analysis showing the overall survival of SGC patients categorized according to whether adjuvant therapy was provided or not**. **(A and B) **Statistical significance of the difference between the curves of the patients showing high and low SPHK1 expression was compared in the non-adjuvant therapy **(A) **and adjuvant therapy **(B) **patient subgroups. **(C and D) **Statistical significance of the difference between the curves of the patients showing same SPHK1 expression was compared in the non-adjuvant therapy **(C) **and adjuvant therapy **(D) **patient subgroups. *P *values were calculated using the log-rank test.

Importantly, when the prognostic value of SPHK1 expression was evaluated in patient subgroups according to treatment (with or without adjuvant therapy), we found that the patients in the group without adjuvant therapy and with tumors exhibiting high SPHK1 expression had significantly lower overall survival rates compared with those with low SPHK1 expression (n = 109; log-rank, *P *< 0.001; Fig. 5A). However, in the adjuvant therapy group, the length of survival time did not differ significantly between the patients with low and high SPHK1 expression (n = 50; log-rank, *P *= 0.695; Fig. 5B). Furthermore, statistical analysis showed that the cumulative 5-year survival rate in the group without adjuvant therapy was 79.9% (95% confidence interval, 0.712-0.891) in the low SPHK1 expression group, whereas it was only 45.1% (95% confidence interval, 0.336-0.566) in the high SPHK1 expression group. Moreover, the prognostic value of similar expression of SPHK1 was evaluated in patient subgroups according to treatment (with or without adjuvant therapy). As shown in Fig. 5C, in the high SPHK1 expression group, the length of survival time did not differ significantly between the patients with or without adjuvant therapy (n = 85; log-rank, P = 0.808; Fig. 5C). Meanwhile, we found that the patients with tumors exhibiting low SPHK1 expression and in the group with adjuvant therapy had lower overall survival rates compared with those without adjuvant therapy (n = 74; log-rank, P = 0.02; Fig. 5D), which suggested that the adjuvant therapy is not suitable to the patient with tumors exhibiting low SPHK1 expression. Taken together, our results suggest that SPHK1 expression might be a novel and valuable predictor for adjuvant therapy to SGC patients.

## Discussion

The key finding of the present study is that the elevated expression of SPHK1 protein is correlated with a poor prognosis and reduced survival of SGC patients, suggesting that SPHK1 is a potential independent prognostic factor for SGC. We found that SPHK1 is upregulated in clinical SGC tissues at both the mRNA and protein levels compared with normal salivary gland tissues. Furthermore, statistical analysis of the immunohistochemical results revealed that the expression level of SPHK1 protein is significantly correlated with SGC clinical characteristics, including clinical stage, TNM classification and pathological differentiation. Moreover, those SGC patients who do not receive adjuvant therapy and who exhibit high SPHK1 expression have a significantly lower overall survival rate compared with those with low SPHK1 expression, indicating that SPHK1 expression might represent a valuable predictor for adjuvant therapy for SGC patients.

Owing to the rarity and histological diversity of SGC, the clinical judgments regarding diagnosis and treatment, as well as the prognosis, always present considerable difficulty [1,2,4,5]. Multiple molecular markers have been shown to be associated with the progression and development of SGC. Several groups have demonstrated that HER2/neu is overexpressed at both the protein and mRNA levels in SGCs, ACCs, and MECs [28-31]. Further studies have shown that the high HER2/neu expressing patients with ACC have a significantly shorter disease-free interval compared to those with low HER2/neu expression [32]. Moreover, the expression of HER-2/neu is correlated with local disease recurrence, distant disease metastasis, and overall survival of different histological types in SGC patients [33-36]. In addition, the expression of mutated tumor suppressor gene *p53 *(in which mutations occur most frequently in exons 7 and 8) has been shown to be associated with the relapse, M classification, and poor prognosis of SDC patients [37]. H-ras mutations, found in various solid tumor types, have also been demonstrated to occur frequently in SGC and are positively correlated with the tumor grade of MECs [38,39]. Moreover, Lequerica-Fernández and colleagues reported that VEGF is upregulated in 62% of SGC tissues. Further, the expression of VEGF is significantly correlated with lymph node metastasis (*P *< 0.005), clinical stage (*P *< 0.02), and disease-specific survival (*P *< 0.01), suggesting that VEGF might contribute to the progression and development of SGC [40]. However, none of these studies established whether these biomarkers could be used as treatment (adjuvant therapy) predictors or indicators to SGC patients. In the current study, we found that the cumulative 5-year survival rate of patients with high SPHK1 expression but without adjuvant therapy was only 45.1% (95% confidence interval, 0.336-0.566). However, it increased to 79.9% (95% confidence interval, 0.712-0.891) in the low SPHK1 expression group even without adjuvant therapy. These results indicate that SPHK1 expression might be a valuable clinical predictor of adjuvant therapy to SGC patients. Furthermore, statistical analysis revealed no difference in the length of survival time between the low and high SPHK1-expressing patients receiving adjuvant therapy, indicating that SPHK1 expression might also represent a valuable clinical indicator of adjuvant therapy to SGC patients. Meanwhile, we found that the patients with tumors exhibiting low SPHK1 expression and in the group with adjuvant therapy had lower overall survival rates compared with those without adjuvant therapy, which suggested that the adjuvant therapy is not suitable to the patient with tumors exhibiting low SPHK1 expression.

Recently, accumulating evidence has suggested that SPHK1 functions as an onco-enzyme that is closely involved in carcinogenesis [20,41,42]. Numerous studies have demonstrated that upregulation of SPHK1 can promote cell proliferation and enhance the resistance to apoptosis induced by different stimuli, and that this upregulation is linked to the failure of clinical cancer therapies, such as chemotherapy and radiotherapy [14,15,18-21]. Consistent with these observations, SPHK1 mRNA and protein levels have been found to be significantly elevated in various tumor types [22-26], which prompted us to ask whether the expression of SPHK1 is upregulated and clinically associated with the progression of SGC. To address this question, we examined the expression of SPHK1 under 3 different circumstances, namely, in normal salivary gland and fresh-frozen SGC tissues, in paired primary SGC tissue and adjacent noncancerous tissue, and in a large cohort of paraffin-embedded SGC tissues. Our data showed that the upregulation of SPHK1 mRNA and protein is a universal and frequent event in human SGC tissues, which indicates that SPHK1 overexpression might be associated with the development and progression of SGC. Importantly, 154 (96.9%) of 159 paraffin-embedded archived SGC specimens, including 9 histological types, exhibited positive staining for SPHK1 in the tumor cells, whereas the adjacent noncancerous cells and normal salivary gland tissue expressed little, if any, SPHK1. Furthermore, statistical analysis of the relationship between SPHK1 staining and the clinicopathological characteristics of the patients revealed a significant correlation between SPHK1 expression and the clinical stage, TNM classification, and pathological differentiation of SGC, further supporting the notion that SPHK1 might play a role in the progression of SGC. It is particularly noteworthy that high SPHK1 expression is associated with a shorter survival time. The cumulative 5-year survival rate was 93.4% (95% confidence interval, 0.854-0.914) in the low SPHK1 expression group, whereas it was only 46.3% (95% confidence interval, 0.369-0.573) in the high SPHK1 expression group, suggesting the possibility that SPHK1 can be used as a predictor for patient prognosis and survival.

## Conclusions

We have demonstrated that the elevated expression of SPHK1 is significantly correlated with the development and progression of SGC. The expression of SPHK1 might represent a novel and potentially independent biomarker for the prognosis of patients with SGC, as well as a novel and valuable predictor for adjuvant therapy to SGC patients. However, in addition to biopsies or surgical tissues, it will be of great interest to investigate whether such an important marker is also detectable in other types of patient samples, such as salivary juice, blood, and oral mucosa. Analysis of such samples would contribute significantly to the detection of SGC at the earliest possible stage, as well as indicating the most appropriate and effective treatment for SGC patients, thereby improving the their chances of recovery and survival.

## Competing interests

The authors declare that they have no competing interests.

## Authors' contributions

GL, HZ and ZZ were responsible for data collection and analysis, executing experiments, interpretation of the results, and writing the manuscript. GL, HZ, ZZ, ZW, and HX, were responsible for conducting the data analysis, reviewing and scoring the degree of immunostaining of sections. JL and LS were responsible for experimental design, analysis and interpretation. All authors have read and approved the final manuscript.

## Pre-publication history

The pre-publication history for this paper can be accessed here:

http://www.biomedcentral.com/1471-2407/10/495/prepub
